# Posterior confluent white matter hyperintensities and intracerebral hemorrhage recurrence risk in patients with cerebral amyloid angiopathy

**DOI:** 10.3389/fneur.2026.1767088

**Published:** 2026-03-13

**Authors:** Jia Liu, Feng Liu, Zihan Wang, Hao Wu, Pan Wang, Hao Lu, Shuai Liu, Shumei Jin, Zhaoyang Lv, Yong Ji, Zhihong Shi

**Affiliations:** 1 Huanhu Hospital Affiliated to Tianjin Medical University, Tianjin, China; 2 Department of Neurology and Tianjin Key Laboratory of Cerebrovascular Disease and Neurodegenerative Disease, Tianjin Dementia Institute, Huanhu Hospital Affiliated to Tianjin Medical University, Tianjin, China; 3 Department of Radiology, Huanhu Hospital Affiliated to Tianjin Medical University, Tianjin, China; 4 Department of Pathology, Huanhu Hospital Affiliated to Tianjin Medical University, Tianjin, China

**Keywords:** cerebral amyloid angiopathy (CAA), intracerebral hemorrhage, magnetic resonance imaging, recurrent, risk factors

## Abstract

**Background:**

Cerebral amyloid angiopathy (CAA) is associated with a high risk of recurrence of intracerebral hemorrhage (ICH). This study aimed to identify risk factors of CAA-related ICH recurrence, especially focused on CAA-characteristic neuroimaging markers.

**Methods:**

Consecutive survivors of spontaneous lobar ICH for probable CAA, possible CAA, and mixed cerebral small vessel disease (CSVD) were enrolled at Tianjin Huanhu Hospital between 2017 and 2024. Baseline clinical data and magnetic resonance imaging (MRI) findings were collected. Posterior confluent white matter hyperintensities (WMH-PC), which means WMHs predominantly posterior to the ventricular horns, extending more than 5 mm in the deep white matter. Other MRI features included multispot white matter hyperintensities (WMH-MS), cortical superficial siderosis (cSS), perivascular spaces (PVS), and acute convexity subarachnoid hemorrhage (cSAH) in the present study. Participants were prospectively followed for recurrent symptomatic ICH or death. Kaplan-Meier and Cox-regression models were used to assess associations with ICH recurrence risk.

**Results:**

The cohort included 254 survivors of spontaneous ICH, with median age of 68.5 years (interquartile range [IQR]: 63.0–76.0) and 63.0% male. WMH-PC was present in 124 patients (48.8%). Hundred and seventy patients (66.9%) were probable CAA-related ICH. Over a median follow-up period of 20.0 months (IQR: 8.0–38.0), 53 patients (20.9%) experienced recurrent ICH. In the probable CAA-related ICH group, WMH-PC (adjusted hazard ratio [aHR]: 2.548; 95% confidence interval [CI]: 1.251–5.193); cSS (aHR: 2.340; 95% CI: 1.126–4.860); and CSO-PVS (aHR: 2.751; 95% CI: 1.219–6.207) were associated with increased risk of ICH recurrence; after adjusted for the MRI features, only CSO-PVS (aHR: 2.278; 95% CI: 1.075–4.828) remained independently associated with an increased risk of ICH recurrence. The effect of combined WMH-PC and cSS on the recurrence of ICH in cases of probable CAA was synergistic (aHR: 3.160; 95% CI: 1.579–6.325).

**Conclusions:**

WMH-PC, cSS and CSO-PVS are risk factors associated with ICH recurrence and WMH-PC in combination with cSS demonstrates a synergistic effect on increased ICH recurrence risk in patients with probable CAA.

## 1 Introduction

Cerebral amyloid angiopathy (CAA) is an age-related cerebral small vessel disease (SVD) characterized by progressive amyloid-β deposition in the media and adventitia of cortical and leptomeningeal arterioles (1, 2). It is the most common cause of spontaneous lobar intracerebral hemorrhage (ICH), particularly in the elderly (3). CAA-related ICH (CAA-ICH) predominantly involves cortical or cortico-subcortical (lobar) regions and is typically characterized by multiple and recurrent hemorrhages (4). Compared with other forms of ICH, CAA-related ICH carries a significantly higher risk of recurrence, reaching up to 10% annually (5, 6).

Recurrent and severe ICH in patients with CAA carries substantial morbidity and mortality, yet its peidictors remain poorly understood. Identified risk predictors for CAA-related ICH include the presence of APOE ε4 and ε2 alleles (7) and the use of anticoagulant or antiplatelet medications (8). Moreover, recent studies and meta-analyses have highlighted CAA-specific magnetic resonance imaging (MRI) markers indicative of vascular injury and white matter damage, including cerebral microbleed (CMB), cortical superficial siderosis (cSS), convexity subarachnoid hemorrhage (cSAH), centrum semiovale perivascular spaces (CSO-PVS), and white matter hyperintensities (WMH), as potential indicators of disease severity (9–11).

WMH are common in aging brains and have been associated with ICH in the general population (12). In patients with CAA, WMH volume has been independently correlated with cerebral amyloid burden as measured by Pittsburgh Compound B retention on positron emission tomography (13). A posterior-predominant pattern of confluent WMH (WMH-PC) has been reported in individuals with lobar ICH (14) and in pathology-confirmed CAA cases without ICH (15). However, the association between WMH-PC and CAA-related ICH recurrence remains inadequately characterized.

This prospective cohort study aimed to investigate risk factors of CAA-related ICH recurrence using an imaging-based approach in patients with hemorrhagic stroke, with particular focus on MRI markers such as WMH-PC, cSS, and CSO-PVS.

## 2 Methods

### 2.1 Ethics

This study was reviewed and approved by the Ethics Committee for Medical Research at Tianjin Huanhu Hospital (NO. 2024–270). Written informed consent was obtained from all participants or their legal representatives before enrollment.

### 2.2 Study population and baseline data collection

This prospective study enrolled adult patients with spontaneous symptomatic lobar intracerebral hemorrhage (ICH) admitted to Tianjin Huanhu Hospital between January 2017 and December 2024. All patients with 3.0-T brain MRI within one week following symptom ICH were enrolled in this study. Diagnosis of probable or possible CAA was established based on histopathology or version 2.0 of the Boston diagnostic criteria (16), whereas mixed cerebral small vessel disease (CSVD) was defined according to the CLAS-ICH (M1-M2) criteria (17). ICH recurrence was defined as a new symptomatic ICH, including both lobar and others (e.g., basal ganglia, thalamus), confirmed by neuroimaging following the index event. All patients underwent 3.0-T brain MRI including T1-weighted, T2-weighted, gradient-echo T2^*^-weighted (GRE-T2^*^), and 3D fluid-attenuated inversion recovery (FLAIR) sequences, with consistent acquisition parameters throughout the study period. Patients were excluded if they did not undergo all of the aforementioned sequences, had unavailable or low-quality imaging, were under the age of 50 years, or had the following conditions: 1) head trauma, 2) cavernous venous malformation, 3) arteriovenous malformation, 4) hemorrhagic cerebral infarction, 5) lobar hemorrhage due to venous sinus thrombosis, or 6) intracranial tumor with hemorrhage (confirmed by imaging or pathology). The patient characteristics clinical findings at the index event were obtained from electronic medical records.

### 2.3 Patient data

Patient information collected at the time of index ICH included age, sex, and medical history of known ICH risk factors including hypertension, diabetes mellitus, coronary artery disease, atrial fibrillation, hyperlipidemia, hyperhomocysteinemia, prior cerebral infarction, stain use, anticoagulant or antiplatelet use, and history of smoking and alcohol consumption.

### 2.4 Imaging analysis

MRI data were independently reviewed by two senior neuroradiologists who were blinded to all clinical and follow-up data, the inter-rater reliability for CAA-related ICH MRI features (WMH-PC/MS, cSS, cSAH, CSO-PVS, CMB) was excellent, with a Cohen's kappa coefficient of 0.85. To minimize confounding by acute hemorrhage, all neuroimaging markers were assessed in the hemisphere contralateral to the index ICH. WMH and perivascular spaces in the centrum semiovale (CSO-PVS) were evaluated on axial T2-weighted and FLAIR sequences. We visually assessed WMH-PC pattern on FLAIR MRI, defined as WMHs predominantly posterior to the ventricular horns, extending more than 5 mm in the deep white matter, with clear separation between ventricular margin (with or without periventricular WMH) (9). Comparison between two trained raters (through review of representative cases and consensus discussions) using 30 cases reflecting the spectrum of WMH-PC pattern presence, and blinded to clinical information, showed excellent interrater agreement (kappa was 0.80). The WMH-multispot pattern (WMH-MS) was defined as more than 10 small, circular or ovoid T2/FLAIR hyperintense lesions in the bilateral subcortical white matter. Severe CSO-PVS was defined as more than 20 visible perivascular spaces in the centrum semiovale of one hemisphere, based on version 2.0 of the Boston criteria (9, 16). WMH severity was graded using the 4-point Fazekas scale (0 = no lesions; 1 = focal; 2 = early confluent; 3 = confluent) (18).

cSS was defined as curvilinear chronic blood products in the subarachnoid space or superficial cortical layers, identified as linear hypointensities on T2^*^GRE, without corresponding hyperintensity on T1-weighted or FLAIR sequences (i.e., excluding acute SAH) (4, 18). Severity of cSS was categorized as focal (≤3 sulci) or disseminated (>3 sulci) (19).

cSAH was defined as acute blood localized to the cortical subarachnoid space, appearing as linear hyperintensity on FLAIR and corresponding hypointensity on T2^*^GRE sequence (20).

CMBs were defined as small, rounded hypointense foci on T2^*^GRE images, primarily located at the cortico-subcortical junction. Strictly lobar CMB typically measure 2–5 mm, but may be up to 10 mm on 3.0-T GRE imaging (21).

### 2.5 Follow-up

Patients who survived for at least 30 days after the index ICH and provided consent for longitudinal follow-up were included. A standardized follow-up procedure was developed: follow-up visits were conducted at 1, 3, 6, and 12 months after discharge, and then once a year subsequently. Follow-up continued until recurrent ICH, death, or the end of the study period (December, 2025). Thirteen (4.8%) patients were lost to follow-up within 1 year, and were excluded as lost-to-follow-up events. A total of 254 survivor patients who were followed up for more than 1 year were included in our study. All follow-up procedures were performed by trained researchers. Follow-up data were obtained from consenting survivors and/or caregivers via hospital readmissions or structured telephone interviews. A unified standard questionnaire was used during the follow-up, with a focus on collecting the following information: whether new neurological symptoms occurred (eg, headache, limb weakness, and disturbance of consciousness), re-examined head CT scan, rebleeding, readmission status, and current survival status.

### 2.6 Statistical analysis

Baseline characteristics of patients with and without recurrent ICH were compared using the χ^2^ test, independent-samples *t*-test, or Mann–Whitney *U*-test, as appropriate. Kaplan-Meier curves were constructed to evaluate ICH recurrence. Survival time was defined as the interval between hospital admission for the index ICH and the time of ICH recurrence, death, or end of follow-up. Univariable Cox proportional hazards models were used to estimate unadjusted hazard ratios (HRs) for ICH recurrence, with MRI-based SVD markers as independent variables. Multivariable Cox regression models adjusted for variables with *p* < 0.05 in univariable analysis (model 1 and model 2). Statistical significance was defined as *p* < 0.05. Analyses were performed using IBM SPSS Statistics, version 26.0 (IBM Corp., Armonk, New York, USA).

## 3 Results

### 3.1 Study participants

**Figure 1:**
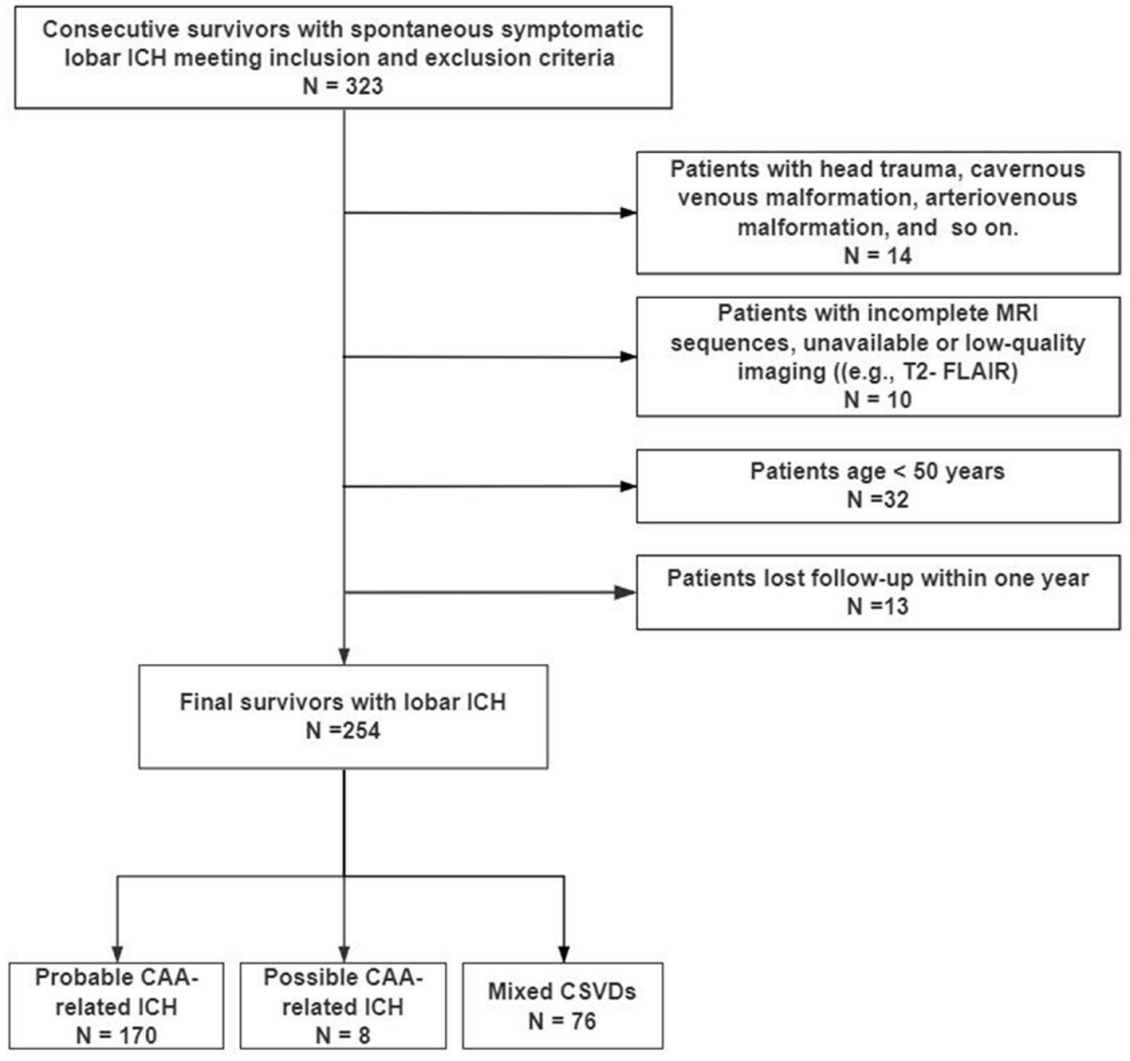
Flow diagram of the study.

The final study cohort comprised 254 survivors of ICH, with a median age of 68.5 years [interquartile range (IQR): 63.0–76.0], of whom 63.0% were male. According to the Boston criteria (version 2.0) (16, 17), the cohort included 170 patients (66.9%) with probable CAA and 42 (16.5%) with possible CAA. Based on the CLAS-ICH criteria (17), 42 patients (16.5%) were diagnosed with mixed CSVD. A flow diagram of participant selection is presented in Figure 1.

During a median follow-up of 20.0 months (interquartile range [IQR]: 8.0–38.0 months), 53 patients (20.9%) experienced recurrent symptomatic ICH, and 26 (10.2%) the recurrent position was in ipsilateral lobe. The median time from the index ICH to first recurrence was 12.0 months (IQR: 4.0–36.0 months). Among patients with recurrent ICH, 34 (64.2%) exhibited WMH-PC on baseline MRI.

### 3.2 Demographics, clinical and neuroimaging characteristics

Among the entire cohort, 124 patients (48.8%) had WMH-PC. Patients with WMH-PC were older than those without (median age: 72.0 vs. 67.0 years; IQR: 65.0–78.0 vs. 59.0–73.0 years; *p* < 0.001). Recurrent ICH occurred more frequently in patients with baseline WMH-PC compared to those without (27.4 vs. 14.6%, *p* = 0.012). cSS was present in 114 patients (44.9%), including 59 (23.2%) with focal cSS and 55 (21.7%) with disseminated cSS. The prevalence of cSS was higher in the WMH-PC group compared to the non-WMH-PC (WMH-NPC) group (49.2 vs. 40.7%, *p* = 0.007). Lobar CMB counts >5 was more common in the WMH-PC group than in the WMH-NPC group (48.4 vs. 25.4%, *p* < 0.001). Similarly, the WMH-PC group had a higher frequency of lacunar infarct (71.0 vs. 43.1%, *p* < 0.001). The finding of WMH-PC was also associated with a higher prevalence of moderate-to-severe (Fazekas grade 2–3) WMH (*p* < 0.001). Detailed cohort characteristics are shown in Table 1.

**Table 1:** Demographic, clinical and neuroimaging characteristics in CAA-ICH patients with posterior confluent WMH and without posterior confluent WMH.

**Characteristics**	**Total ICH patients (*n =* 254)**	**Patients without WMH-PC (*n =* 130)**	**Patients with WMH-PC (*n =* 124)**	***p*-value**
Gender, male, *n* (%)	160 (63.0%)	78 (60.0%)	82 (66.1%)	0.312
Age, (media, IQR)	68.5 (63.0–76.0)	67.0 (59.0–73.0)	72.0 (65.0–78.0)	**< 0.001** ^**^
Smoking, *n* (%)	102 (40.2%)	50 (38.5%)	52 (41.9%)	0.572
Drinking, *n* (%)	73 (28.7%)	35 (26.9%)	38 (30.6%)	0.512
**Clinical characteristics**	
Hypertension, *n* (%)	141 (55.5%)	69 (53.1%)	72 (58.1%)	0.424
Diabetes mellitus, *n* (%)	46 (18.1%)	24 (18.5%)	22 (17.7%)	0.882
CAD, *n* (%)	41 (16.1%)	23 (17.7%)	18 (14.5%)	0.492
Atrial fibrillation, *n* (%)	12 (4.7%)	6 (4.6%)	6 (4.8%)	0.933
Cerebral infarction, *n* (%)	62 (24.4%)	29 (22.3%)	33 (26.6%)	0.425
Hyperlipidemia, *n* (%)	139 (54.7%)	78 (60.0%)	61 (49.2%)	0.084
Hyperhomocysteinemia, *n* (%)	24 (9.4%)	7 (5.4%)	17 (13.7%)	**0.023** ^*^
Anticoagulant medicine, *n* (%)	9 (3.5%)	5 (3.8%)	4 (3.2%)	0.789
Antiplatelet medicine, *n* (%)	45 (17.7%)	23 (17.7%)	22 (17.7%)	0.992
Statin use, *n* (%)	48 (18.9%)	25 (19.2%)	23 (18.5%)	0.890
ICH recurrent, *n* (%)	53 (20.9%)	19 (14.6%)	34 (27.4%)	**0.012** ^*^
**Neuroimaging characteristics**	
cSAH, *n* (%)	52 (20.5%)	32 (24.6%)	20 (16.1%)	0.094
SDH, *n* (%)	48 (18.9%)	26 (20.0%)	22 (17.7%)	0.646
cSS, *n* (%)				**0.007** ^**^
Focal cSS	59 (23.2%)	35 (26.9%)	24 (19.4%)	
Disseminated cSS	55 (21.7%)	18 (13.8%)	37 (29.8%)	
WMH-MS, *n* (%)	112 (44.1%)	53 (40.8%)	59 (47.6%)	0.274
Severe WMH, Fazekas score 2–3, *n* (%)	150 (59.1%)	45 (34.6%)	105 (84.7%)	**< 0.001** ^**^
CSO-PVS, *n* (%)	134 (52.8%)	62 (47.7%)	72 (58.1%)	0.098
Lobar CMBs (>5), *n* (%)	93 (36.6%)	33 (25.4%)	60 (48.4%)	**< 0.001** ^**^
Lacunar infarction, *n* (%)	144 (56.7%)	56 (43.1%)	88 (71.0%)	**< 0.001** ^**^

Significant differences are marked with ^*^p < 0.05, ^**^p < 0.01. Bold values indicate statistical significance (*p* < 0.05). CAA, cerebral amyloid angiopathy; CAD, coronary artery disease; cSAH, convexity subarachnoid hemorrhage; SDH, subdural hematoma; ICH, intracerebral hemorrhage; WMH, white matter hyperintensities; WMH-MS, multispot white matter hyperintensities; WMH-PC, posterior confluent white matter hyperintensities; cSS, cortical superficial siderosis; CSO-PVS, centrum semiovale perivascular space; CMB, cerebral microbleed; IQR, interquartile range; MRI, magnetic resonance imaging.

### 3.3. Comparison of patients with vs. without ICH recurrence

The presence of WMH-PC and cSS was significantly associated with recurrent symptomatic ICH within the cohort (*p* = 0.012 and *p* = 0.006, respectively). Compared to those without recurrence, patients with recurrent ICH were older at the index event and more frequently exhibited findings of cSAH, SDH, IVH, WMH-MS, CSO-PVS, lobar CMB, and lacunar infarct, although these associations did not reach statistical significance. History of hypertension, diabetes mellitus, coronary heart disease (CHD), atrial fibrillation, prior cerebral infarction, smoking, alcohol use, and anticoagulant or antiplatelet agent use were not significantly associated with ICH recurrence in this group (*p* >0.05). Baseline characteristics stratified by recurrence status are detailed in Table 2.

**Table 2:** Characteristics of patients with vs. without ICH recurrence during follow-up.

**Characteristics**	**Patients without recurrent ICH (*n =* 201)**	**Patients with recurrent ICH (*n =* 53)**	***p*-value**
Gender, male, *n* (%)	129 (64.2%)	31 (58.5%)	0.445
Age, (media, IQR)	68.0 (61.0–76.0)	70.0 (64.0–77.0)	0.199
Smoking, *n* (%)	84 (41.8%)	18 (34.0%)	0.301
Drinking, *n* (%)	60 (29.9%)	13 (24.5%)	0.446
**Clinical characteristics**	
Hypertension, *n* (%)	113 (56.2%)	28 (52.8%)	0.659
Diabetes mellitus, *n* (%)	40 (19.9%)	6 (11.3%)	0.149
CAD, *n* (%)	34 (16.9%)	7 (13.2%)	0.514
Atrial fibrillation, *n* (%)	11 (5.5%)	1 (1.9%)	0.274
Cerebral infarction, *n* (%)	52 (25.9%)	10 (18.9%)	0.291
Hyperlipidemia, *n* (%)	109 (54.2%)	30 (56.6%)	0.757
Hyperhomocysteinemia, *n* (%)	19 (9.5%)	5 (9.4%)	0.997
Anticoagulant medicine, *n* (%)	7 (3.5%)	2 (3.8%)	0.919
Antiplatelet medicine, *n* (%)	39 (19.4%)	6 (11.3%)	0.170
Statin use, *n* (%)	40 (19.9%)	8 (15.1%)	0.427
**Neuroimaging characteristics**	
cSAH, *n* (%)	37 (18.4%)	15 (28.3%)	0.112
SDH, *n* (%)	34 (16.9%)	14 (26.4%)	0.116
cSS, *n* (%)			**0.006** ^**^
Focal cSS	42 (20.9%)	17 (32.1%)	
Disseminated cSS	38 (18.9%)	17 (32.1%)	
WMH-MS, *n* (%)	88 (43.8%)	24 (45.3%)	0.845
WMH-PC, *n* (%)	90 (44.8%)	34 (64.2%)	**0.012** ^*^
Severe (Fazekas score 2–3) WMH, *n* (%)	116 (57.7%)	34 (64.2%)	0.396
CSO-PVS, *n* (%)	102 (50.7%)	32 (60.4%)	0.212
Lobar CMBs (>5), *n* (%)	68 (33.8%)	25 (47.2%)	0.073
Lacunar infarction, *n* (%)	111 (55.2%)	33 (62.3%)	0.357

Significant differences are marked with ^*^p < 0.05, ^**^p < 0.01. Bold values indicate statistical significance (*p* < 0.05). CAD, coronary artery disease; cSAH, convexity subarachnoid hemorrhage; SDH, subdural hematoma; ICH, intracerebral hemorrhage; WMH, white matter hyperintensities; WMH-MS, multisport white matter hyperintensities; WMH-PC, posterior confluent white matter hyperintensities; cSS, cortical superficial siderosis; CSO-PVS, centrum semiovale perivascular space; CMB, cerebral microbleed; IQR, interquartile range.

### 3.4 Neuroimaging characteristics of probable CAA, and mixed CSVD

The neuroimaging characteristics of probable CAA, and mixed CSVD are summarized in Table 3. There were significant intergroup differences between the groups in the prevalence of cSAH (*p* = 0.040), cSS (*p* = 0.019), lobar CMBs (≥1; *p* = 0.001), lobar CMBs (>5; *p* = 0.001), WMH-MS (*p* = 0.035), CSO-PVS (*p* = 0.003), severe (Fazekas score 2–3) WMH (*p* = 0.004), and lacunar infarction (*p* < 0.001). Compared with the mixed CSVD groups, the probable CAA group showed higher frequencies of cSAH, cSS, CSO-PVS, as well as larger hemorrhage volumes. However, the mixed CSVD-ICH group showed obvious sever Fazekas score (2, 3), and higher percentage of lobar CMBs.

**Table 3:** Neuroimaging characteristics in probable CAA and mixed cerebral small vessel disease.

**Characteristics**	**Probable CAA (*n =* 170)**	**Mixed cerebral small vessel disease (*n =* 76)**	***p*-value**
cSAH	42 (24.7%)	10 (12.2%)	**0.040** ^*^
cSS, *n* (%)			**0.019** ^*^
Focal cSS	47 (27.6%)	12 (15.8%)	
Disseminated cSS	39 (22.9%)	16 (21.1%)	
Lobar CMB counts (≥1), *n* (%)	74 (43.5%)	49 (64.5%)	**0.001** ^*^
Lobar CMB counts (>5), *n* (%)	45 (26.5%)	36 (47.4%)	**0.001** ^*^
Deep CMBs, *n* (%)	0	70 (92.0%)	
CSO-PVS	98 (57.6%)	36 (47.4%)	**0.003** ^*^
WMH-MS, *n* (%)	79 (46.5%)	33 (43.4%)	**0.035** ^*^
WMH-PC, *n* (%)	76 (44.7%)	43 (56.6%)	0.167
Severe (Fazekas score 2–3) WMH	86 (50.6%)	55 (72.4%)	**0.004** ^**^
Chronic/macro-hemorrhages	19 (11.2%)	9 (9.2%)	0.559
Recurrent ICH	39 (22.9%)	14 (18.4%)	0.243
Recurrent ICH position			0.132
Ipsilateral lobe	21 (12.4%)	2 (2.6%)	
Contralateral lobe	10 (5.9%)	2 (2.6%)	
Deep	4 (2.4%)	7 (9.2%)	
Unknown position	4 (2.4%)	3 (3.8%)	
Lacunar infarction, *n* (%)	9 (58.2%)	63 (82.9%)	**0.001** ^**^
Hemorrhage volume (median, IQR)	16.0 (7.0–36.5)	14.0 (6.0–31.0)	0.150

Significant differences are marked with ^*^p < 0.05, ^**^p < 0.01. Bold values indicate statistical significance (*p* < 0.05). cSAH, subarachnoid hemorrhage; WMH-MS, multispot white matter hyperintensities; WMH-PC, posterior confluent white matter hyperintensities; CMB, cerebral microbleed; cSS, cortical superficial siderosis; CSO-PVS, centrum semiovale perivascular space; IQR, interquartile range.

### 3.5. Survival analysis

#### 3.5.1 Kaplan-Meier curves for ICH recurrence according to the presence of WMH-PC

**Figure 2:**
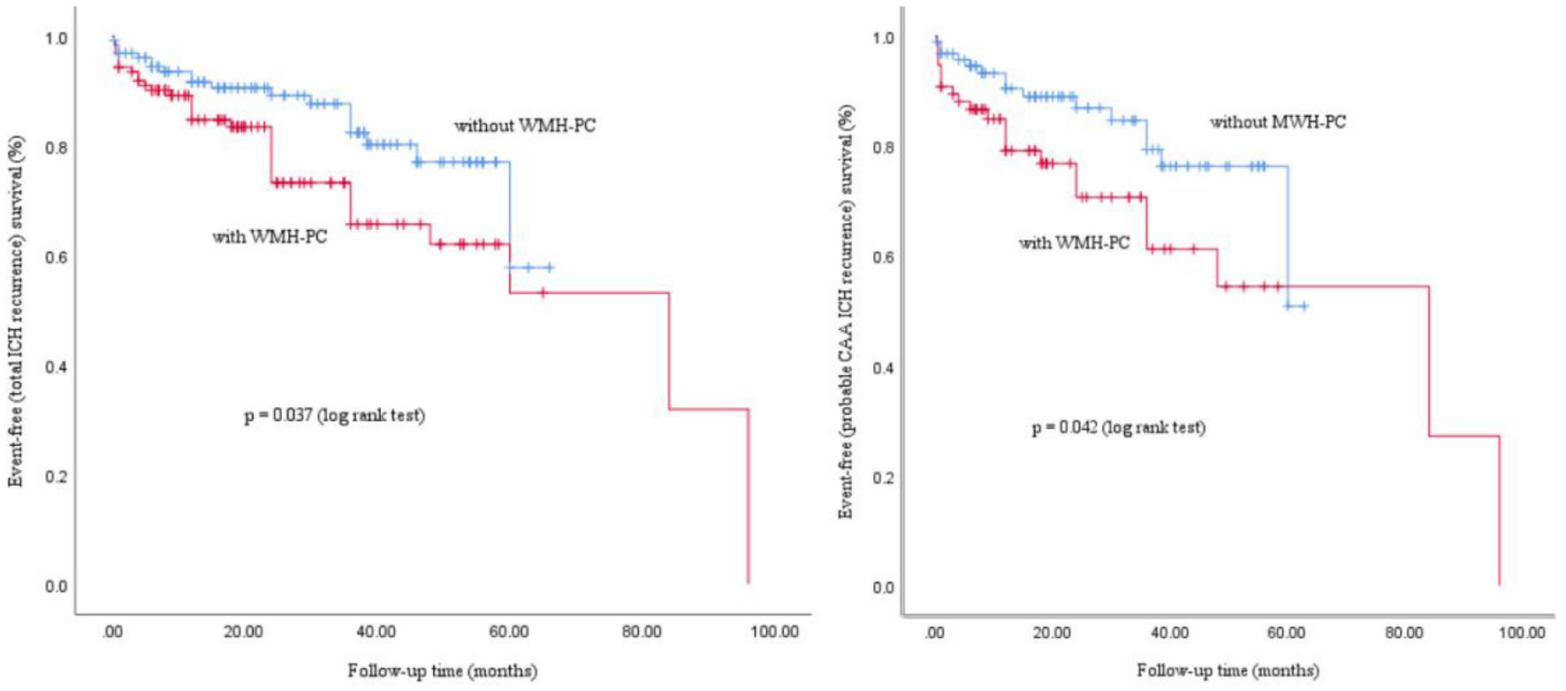
Kaplan-Meier curves for ICH recurrence according to the presence of WMH-PC on MRI.

Kaplan-Meier analysis demonstrated that in total lobar ICH and in probable CAA-ICH, patients without WMH-PC had a significantly higher ICH recurrence-free survival probability than those with WMH-PC (log-rank *p* = 0.037 and *p* = 0.042, respectively), also supporting the presence of WMH-PC as a risk factor of recurrent ICH (Figure 2).

#### 3.5.2 Cox regression analysis for ICH recurrence according to the presence of WMH-PC

In the total ICH group, the univariable Cox regression analysis identified several variables that were significantly associated with increased risk of ICH recurrence, including WMH-PC ([HR]: 1.826; 95% [CI]: 1.021–3.268), focal cSS (HR: 2.432; 95% CI: 1.257–4.706), disseminated cSS (HR: 2.033; 95% CI: 1.045–3.956), cSAH (HR: 1.967; 95% CI: 1.068–3.622), the combination of cSS and WMH-PC (HR: 2.730; 95% CI: 1.555–4.796), and the combination of cSS and CSO-PVS (HR: 2.299; 95% CI: 1.307–4.044). Two multivariable Cox regression models were conducted to evaluate the independent predictive value of MRI features. Model 1 which adjusted for clinical confounders (e.g, smoking, hypertension, hyperlipidemia, hyperhomocysteinemia, and history of statin, antiplatelet or anticoagulant medication use), showed the presence of WMH-PC (aHR: 2.132; 95% CI: 1.179–3.855), cSS (aHR: 2.109; 95% CI: 1.185–3.755), the combination of cSS and WMH-PC (aHR: 2.943; 95% CI: 1.657–5.226), and the combination of cSS and CSO-PVS (aHR: 2.134; 95% CI: 1.190–3.827) remained independently associated with an increased risk of ICH recurrence during the follow-up period. Model 2 which adjusted for the MRI features with significant associations in univariable analysis, including cSAH and cSS, showed the presence of WMH-PC (aHR: 1.888; 95% CI: 1.044–3.414), cSS (aHR: 2.042; 95% CI: 1.154–3.612), and cSAH (aHR: 2.116; 95% CI: 1.135–3.944) remained independently associated with an increased risk of ICH recurrence (Table 4).

**Table 4:** Univariable and multivariable Cox regression analyses for predictors of recurrent ICH in total ICH and probable CAA.

**Variables**	**Total ICH**			**Probable CAA**		
	**Univariable analysis**	**Multivariate analysis model 1**	**Multivariate analysis model 2**	**Univariable analysis**	**Multivariate analysis model 1**	**Multivariate analysis model 2**
	**HR (95% CI)**	**Adjusted-HR (95% CI)**	**Adjusted-HR (95% CI)**	**HR (95% CI)**	**Adjusted-HR (95% CI)**	**Adjusted-HR (95% CI)**
cSAH	**1.967 (1.068–3.622)** ^*^	1.842 (0.994–3.414)	**2.116 (1.135–3.944)** ^*^	1.934 (0.974–3.841)	1.790 (0.895–3.580)	—
cSS	**2.218 (1.256–3.916)**	**2.109 (1.185–3.755)**	**2.042 (1.154–3.612)** ^*^	**2.313 (1.131–4.278)**	**2.340 (1.126–4.860)**	1.869 (0.890–3.921)
CSO-PVS	1.555 (0.892–2.711)	1.710 (0.941–3.110)	—	**2.609 (1.240**–5.489)^*^	**2.751 (1.219**–6.207)^*^	**2.278 (1.075–4.828)** ^*^
WMH-PC	**1.826 (1.021–3.268)** ^*^	**2.132 (1.179–3.855)** ^*^	**1.888 (1.044–3.414)** ^*^	**1.965 (1.002**–3.852)^*^	**2.548 (1.251**–5.193)^*^	1.500 (0.747–3.015)
WMH-MS	0.779 (0.435–1.395)	0.742 (0.409–1.345)	—	0.703 (0.355–1.392)	0.788 (0.391–1.587)	—
cSS and WMH-PC	**2.730 (1.555–4.796)** ^**^	**2.943 (1.657–5.226)** ^**^	—	**2.633 (1.350–5.132)** ^**^	**3.160 (1.579–6.325)** ^**^	—
cSS and CSO-PVS	**2.299 (1.307–4.044)** ^*^	**2.134 (1.190–3.827)** ^*^	—	**2.313 (1.210**–4.420)^**^	**2.044 (1.016–4.115)** ^*^	—

Significant differences are marked with ^*^p < 0.05, ^**^p < 0.01. Bold values indicate statistical significance (*p* < 0.05). Univariable cox proportional hazards models. Multivariable Cox regression model 1 prefers to adjusting for clinical confounders (smoking, hypertension, hyperlipidemia, hyperhomocysteinemia, and history of statin, antiplatelet or anticoagulant medication use). Multivariable cox regression model 2 prefers to adjusting for MRI features. cSAH, convexity subarachnoid hemorrhage; cSS, cortical superficial siderosis; WMH-PC, posterior confluent white matter hyperintensities; WMH-MS, multispot white matter hyperintensities; CSO-PVS, centrum semiovale perivascular space; HR, hazard ratio.

In the probable CAA group, the univariable Cox regression analysis identified several neuroimaging markers, including: WMH-PC (HR: 1.965; 95% CI: 1.002–3.852), cSS (HR: 2.313; 95% CI: 1.131–4.278), and CSO-PVS (HR: 2.609; 95% CI: 1.240–5.489), as significantly associated with an increased risk of recurrent ICH. Additionally, the combined presence of cSS with WMH-PC (HR: 2.633; 95% CI: 1.350–5.132), and cSS with CSO-PVS (HR: 2.313; 95% CI: 1.210–4.420), also showed significant associations with an increased risk of recurrent ICH. In the multivariable Cox regression model 1, adjusting for clinical confounders, findings of WMH-PC (aHR: 2.548; 95% CI: 1.251–5.193), cSS (aHR: 2.340; 95% CI: 1.126–4.860), CSO-PVS (aHR: 2.751; 95% CI: 1.219–6.207), the combined cSS with WMH-PC (aHR: 3.160; 95% CI: 1.579–6.325), and the combination of cSS and CSO-PVS (aHR: 2.044; 95% CI: 1.016–4.115) remained independently associated with an increased risk of ICH recurrence during the follow-up period. However, in model 2 incorporating MRI features, only CSO-PVS (aHR: 2.278; 95% CI: 1.075–4.828) remained independently associated with an increased risk of ICH recurrence, WMH-PC was not an independent factor associated with recurrent ICH (Table 4).

## 4 Discussion

Among all 254 survivors of spontaneous ICH, 53 patients (20.9%) experienced recurrent ICH. In probable CAA-related group, the presence of WMH-PC, cSS and CSO-PVS are risk factors associated with ICH recurrence. Furthermore, the combined effect of the coexistence of WMH-PC and cSS on ICH recurrence risk was greater than that of the individual effects in cases of total lobar ICH and probable CAA-related ICH.

In this study, WMH-PC was an importent risk factor of recurrent ICH. It was significantly associated with total lobar ICH (aHR = 2.132), and this association was even more pronounced in the probable CAA subgroup (aHR = 2.548). The recurrenec of ICH in CAA had temporal and spatial clustering (22). Previous research has shown that increased WMH burden is correlated with ICH volume and hematoma growth (23, 24), but without apparent association with recurrent cerebral hemorrhage. Until now, few studies have reported that a posterior distribution of WMH can also independently predict recurrent ICH in patients with CAA. WMH in the centrum semiovale and posterior brain regions are particularly associated with CAA pathology (18, 24). Pathophysiologically, WMH may reflect chronic ischemia, white matter rarefaction, disruption of the blood-brain barrier (BBB), and reduced vascular integrity (25), and WMH severity correlates with several disease markers, including the vascular amyloid-β burden (26), the CMB count (9), the lobar ICH risk (27), plasma amyloid-β 40 levels (28), and the risk of cognitive decline (29). A recent combined *in vivo* MRI-*ex vivo* MRI-neuropathological study has identified a correlation between vascular Aβ severity and WMH volume in definite CAA (26). Histopathological data reveal that posterior confluence of WMH can also indicate white matter rarefaction, chronic neuroinflammation, and advanced arteriolosclerosis (26, 30). These findings underscore the importance of WMH-PC as a marker of disease severity and recurrence risk in CAA-ICH.

The findings regarding cSS are consistent with those of previous studies (18, 31, 32) that identified cSS (specially disseminated cSS) as an independent and robust predictor of ICH recurrence in patients with CAA. One recent study showed that MRI-based subtypes of ICH that the highest recurrence risk was found in CAA, second was found in mixed CSVD, and cSS was an independent risk factors on the recurrence of CAA-related ICH (33). Other evidence have confirmed that cSS significantly increases the risk of symptomatic lobar ICH (6, 34, 35), and meta-analysis further support this association (11, 36). In the present study, WMH-PC was not independently associated with the presence of CSS on ICH recurrence. The combination of cSS and WMH-PC exhibited a strong synergistic effect, representing a high risk of recurrence in both total ICH (aHR = 2.943) and probable CAA (aHR = 3.160). The presence of cSS has been proposed as an indicator of high early disease activity (4), whereas the finding of WMH-PC corresponds to chronic vascular compromise. The coexistence of these two imaging markers suggests a state of severe, persistent small vessel dysfunction that could substantially increase the risk of recurrent fatal hemorrhage in the long term.

This study also identified CSO-PVS as an independent risk factor of recurrent ICH in patients with probable CAA. The Boston criteria v2.0 contends CSO-PVS as a new non-hemorrhagic marker of CAA % (37). In a large single-center cohort of survivors with CAA-related ICH, as well as in a retrospective case-control study, a higher burden of CSO-PVS was found to be associated with a greater risk of ICH recurrence (31, 34). The result further support the importance of specific neuroimaging markers over generalized SVD burden in guiding clinical management.

In our probable CAA group, the rate of lacunar infarction was higher than that of CMBs (26%). As shown in the previous article (38), the lacunar infarction in the cerebral lobar of patients with CAA is significantly positively correlated with CMBs. This study also confirmed that the prevalence of deep lacunar infarction is close to that of hypertensive intracerebral hemorrhage. The mixed CSVD-ICH group showed higher percentage lobar CMBs than probable CAA-ICH patients in our study. This was consistent with previous study results: hypertensive CSVD is the predominant microangiopathy with a combination of lobar and deep cerebral microbleeds (CMBs) and intracerebral hemorrhage (mixed ICH), mixed ICH with cSS likely includes both hypertensive cerebral small vessel disease (HTN-cSVD) and CAA, whereas mixed ICH without cSS is likely driven by HTN-cSVD. CSS is a marker strongly associated with CAA (39).

Several limitations should be noted. First, this was a single-center study, which may limit generalizability. However, the large sample size and extended follow-up help mitigate this concern. Second, we were unable to account for variations in blood pressure control or medication use (e.g., antihypertensives, anticoagulants, or antiplatelet agents) during follow-up period, which could influence recurrence risk. Nevertheless, the primary objective was to assess the predictive value of neuroimaging markers such as WMH-PC rather than identify all possible contributors to ICH recurrence. Finally, it should be emphasized that differences in WMH distribution may not be fully captured by visual inspection alone.

## 5 Conclusions

In conclusion, this study demonstrates that WMH-PC, cSS and CSO-PVS are risk factors associated with CAA-related ICH recurrence. In addition, the combination of WMH-PC and cSS demonstrates a synergistic effect on increased ICH recurrence risk in patients with probable CAA. These findings enhance the current understanding of the CAA neuroimaging profile and its association with clinical outcomes. Furthermore, they may inform future therapeutic strategies and aid in risk stratification to prevent recurrence in this vulnerable population.

## Data Availability

The raw data supporting the conclusions of this article will be made available by the authors, without undue reservation.
